# Geographic Variation in Access to Dog-Bite Care in Pakistan and Risk of Dog-Bite Exposure in Karachi: Prospective Surveillance Using a Low-Cost Mobile Phone System

**DOI:** 10.1371/journal.pntd.0002574

**Published:** 2013-12-12

**Authors:** Syed Mohammad Asad Zaidi, Alain B. Labrique, Saira Khowaja, Ismat Lotia-Farrukh, Julia Irani, Naseem Salahuddin, Aamir Javed Khan

**Affiliations:** 1 Interactive Research and Development, Karachi, Pakistan; 2 Department of International Health, Johns Hopkins Bloomberg School of Public Health, Baltimore, Maryland, United States of America; 3 Center for International Health, University of Bergen, Bergen, Norway; 4 Department of Infectious Diseases, Indus Hospital, Karachi, Pakistan; The Global Alliance for Rabies Control, United States of America

## Abstract

**Background:**

Dog-bites and rabies are under-reported in developing countries such as Pakistan and there is a poor understanding of the disease burden. We prospectively collected data utilizing mobile phones for dog-bite and rabies surveillance across nine emergency rooms (ER) in Pakistan, recording patient health-seeking behaviors, access to care and analyzed spatial distribution of cases from Karachi.

**Methodology and Principal Findings:**

A total of 6212 dog-bite cases were identified over two years starting in February 2009 with largest number reported from Karachi (59.7%), followed by Peshawar (13.1%) and Hyderabad (11.4%). Severity of dog-bites was assessed using the WHO classification. Forty percent of patients had Category I (least severe) bites, 28.1% had Category II bites and 31.9% had Category III (most severe bites). Patients visiting a large public hospital ER in Karachi were least likely to seek immediate healthcare at non-medical facilities (Odds Ratio = 0.20, 95% CI 0.17–0.23, p-value<0.01), and had shorter mean travel time to emergency rooms, adjusted for age and gender (32.78 min, 95% CI 31.82–33.78, p-value<0.01) than patients visiting hospitals in smaller cities. Spatial analysis of dog-bites in Karachi suggested clustering of cases (Moran's I = 0.02, p value<0.01), and increased risk of exposure in particular around Korangi and Malir that are adjacent to the city's largest abattoir in Landhi. The direct cost of operating the mHealth surveillance system was USD 7.15 per dog-bite case reported, or approximately USD 44,408 over two years.

**Conclusions:**

Our findings suggest significant differences in access to care and health-seeking behaviors in Pakistan following dog-bites. The distribution of cases in Karachi was suggestive of clustering of cases that could guide targeted disease-control efforts in the city. Mobile phone technologies for health (mHealth) allowed for the operation of a national-level disease reporting and surveillance system at a low cost.

## Author Summary

Resource constraints prevent adequate surveillance of neglected infectious diseases such as rabies in developing countries leading to a poor understanding of the disease burden and limited evidence with which to design effective control measures. We utilized a low cost mobile-phone based system to carry out the first prospective surveillance of dog-bites and rabies in Pakistan by screening all patients presenting to nine emergency rooms in eight cities over a two-year period. We found a large number of dog-bite cases (nearly a third of which were severe based on a World Health Organization classification) with substantial geographical variability in time to presentation as well as health-seeking behavior following dog-bites across the reporting sites. Spatial analyses of collected data from Karachi, Pakistan's largest city identified areas with increased risk of dog-bite exposure, which has implications for the design of necessary control measures such as dog vaccination. While mobile phone based technologies have the potential to address limitations in disease surveillance in developing countries, the cost-effectiveness of large scale implementations of such strategies need to be explored and further evaluated where appropriate.

## Introduction

Infectious disease surveillance continues to remain challenging in developing countries with resource constraints, weak health systems and poor reporting mechanisms [1], [2]. Existing limitations in achieving these core capacities of the International Health Regulations (IHR) have been further compounded in Pakistan by the closure of the Ministry of Health in 2011 and devolution of some of its roles to the provinces, which has disrupted central information collection and dissemination processes [3], [4]. Donor resources for surveillance are currently dedicated towards certain high priority programs such as active surveillance for acute flaccid paralysis under the polio eradication initiative, while surveillance for other endemic or emerging infectious diseases has been given far less attention. Determining a more accurate burden of these less-studied illnesses is necessary to design appropriate preventative measures and to establish best clinical practice. Recent innovations in mobile phone technologies and the rapid growth of the telecommunications sector in developing countries like Pakistan provide possible solutions to filling this knowledge gap.

Rabies is a notifiable disease in most developed countries; however, cases are generally underreported in countries like Pakistan and there is a poor understanding of the disease burden [5]. South Asia is one of the few regions of the world where the epidemiology of rabies is driven through the urban cycle (primary transmission of the virus occurs through dog-bites rather than wildlife), even though effective control and preventative measures for the disease have long been established [6]. In resource-constrained settings, high-risk areas need to be identified to target interventions for effective rabies control and elimination. In addition, gaps need to be identified in clinical and public health practice where appropriate preventative treatment is either delayed or is inadequate following dog-bites. Routine surveillance of dog-bites and rabies in Pakistan is currently conducted under the government's HMIS (Health Management Information System) reporting program but poor quality of collected data prevents evidence-based disease control efforts. Published journal articles on the other hand have relied upon retrospectively collected data from hospital records [7]–[9] and may not adequately capture information regarding key rabies prevention measures recommended by the WHO or to guide efforts towards local canine rabies elimination [10]. We present an analysis of prospectively collected data utilizing mobile phone based health technologies, or mHealth, for dog-bite and rabies surveillance across nine sites in Pakistan, with technical and financial support from the World Health Organization (WHO).

## Methods

### Study Objectives

The aims of this study were to: 1) estimate the burden of dog-bites in Pakistan; 2) describe the frequency, age and gender distributions and severity of dog-bite cases across the reporting sites; 3) assess differences in patient health-seeking behavior and patient access to care amongst the reporting sites; 4) identify high-risk neighborhoods for dog-bite exposure in Karachi, Pakistan's most populous city; 5) describe implementation costs of the mHealth surveillance system from a health services perspective.

### Study Sites

**Figure 1:**
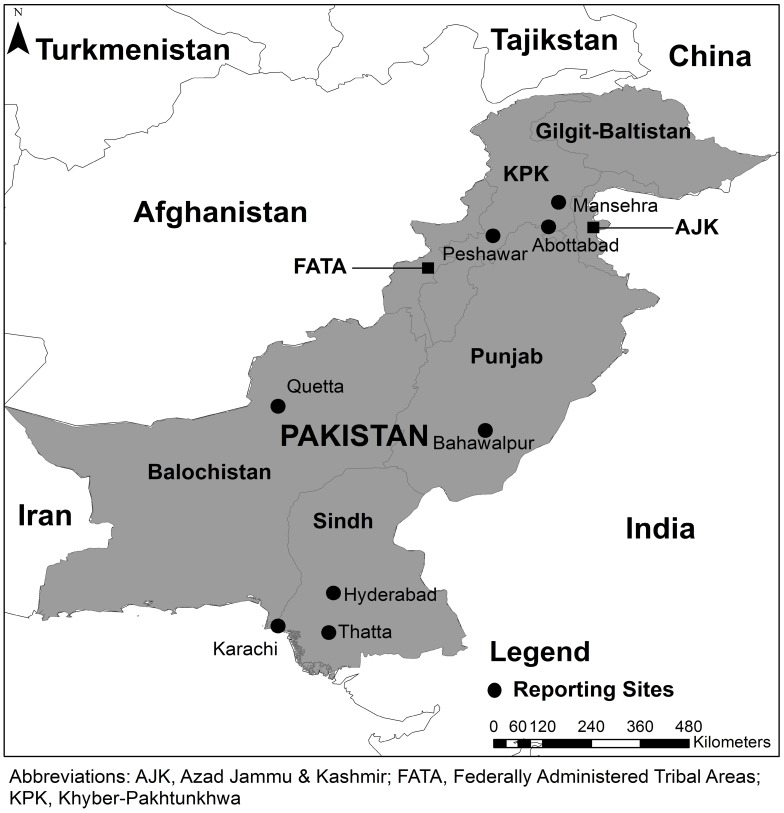
Phone based screening for dog-bites and rabies was established at nine tertiary hospital emergency rooms across Pakistan, including 2 in Karachi, February 2009–February 2011.

Surveillance was carried out from February 2009 to February 2011. Figure 1 shows the locations of participating Emergency Rooms (ER) in Hyderabad, Thatta, Bahawalpur, Abbottabad, Peshawar, Mansehra, Quetta and two sites in Karachi (one public and one private). ERs were based in high volume tertiary care hospitals that were well known as major referral hospitals for dog-bites and provided rabies vaccination, either the sheep-brain tissue vaccine from the government or cell-culture vaccine from philanthropic donors. The hospital ERs served as reporting centers for the study. Several other public and private facilities providing emergency medical care exist in these cities, particularly in Karachi. For the remaining cities, the selected hospitals were the primary facilities for emergency care serving a mix of urban and rural populations. Reporting from two centers in Lahore and Rawalpindi ceased during the course of the study due to deteriorating security at the time and poor institutional support for surveillance. Limited data was collected from these centers and is not included in the present analysis.

### Patient Selection

Cases were defined as all patients presenting with dog-bites to emergency rooms at enlisted centers and patients with dog-bites referred from other facilities. If dog-bite victims did not seek healthcare, or sought care with traditional healthcare providers and alternative medical centers from where referrals were not made to an enlisted center, those cases would not be reported through our system. In addition, information on some cases was not captured because of refusals to take part in the questionnaire and could not be included.

### Recruitment of Screeners

Lay persons with at least 12 years of education were recruited on recommendation of local hospital administrators, and were provided training for the role of “screener” to implement the study protocols regarding informed consent procedures and phone-based screening. The training emphasized basic research ethics and the necessity of voluntary informed consent. Training was provided onsite for data entry and storage using the mobile phone and included role-play to enable screeners to familiarize themselves with the study tools. The administrative heads of ERs were involved in supervision of screeners and in providing local logistical support. Screeners were provided a monthly stipend of approximately USD 80 per month.

### Data Collection

Dog-bite cases were approached immediately after initial treatment, introduced to the study and its objectives and consent was requested for an interview. A standardized questionnaire using a mobile phone based application was completed for all consenting patients. The questionnaire was created using the openXdata software package for low-cost mobile devices. OpenXdata is an open source software for service providers and researchers to design and manage forms for data collection using mobile devices and remotely monitor data collection activity in the field. OpenXdata has two components, a web-based server application and openXdata mobile which is installed on low cost phones. We provided a GPS-enabled Nokia 6220 mobile phone (costing USD 250 each) to screeners at each site programmed with forms to collect patient demographics, details about the nature of the bite and geographic location of the incident. A patient's attendants provided history of exposure and treatment in cases where rabies was suspected and the patient was incapable of responding. The use of openXdata allowed for predefined validation checks as data was being entered into the phone, and eliminated the use of paper forms other than for consent. Date and time stamps were automatically recorded along with GPS coordinates each morning immediately outside the site to monitor the screeners attendance at the hospital. All data entered via mobile phones was uploaded on to the centralized openXdata server, using a GPRS connection.

### Ethical Consent

Ethical approval for the study was obtained from the Institutional Review Board (IRB) at Interactive Research & Development (IRD) which is registered with the U.S. Department of Health and Human Services (DHHS), Office for Human Research Protections (IRB # 00005148). Informed consent was obtained from patients or from guardians in the case of children, through signatures or thumbprints (for illiterate patients) onto paper consent forms, each of which was bar-coded with the patient ID to enable linking with electronic forms. Approval for the use of thumb ink prints in patients unable to provide signed consent as well as for guardians in the case of children was provided by the IRB. An IRB exemption was subsequently obtained for retrospective analysis of surveillance data following closure of the study and data collection activities.

### Statistical Analysis

Statistical analysis was carried out using Stata version 12.1 (College Station, TX: StataCorp LP). We calculated summary statistics by center for total number of cases, patient demographics and severity of bites based on the WHO classification [10]. Type of care immediately sought after the bite, included visits to hospitals, general practitioners, homeopathic facilities, spiritual healers or self-treatment at home. These were categorized as a binary variable to compare treatment at a medical facility versus treatment at a non-medical facility and administering self-treatment. Summary statistics by center were calculated for type of immediate care sought after dog-bites and patient travel time to emergency room. We subsequently fitted a multiple logistic regression to the binary variable as a measure of assessing differences in health-seeking behavior with reporting center, age (centered at the mean) and gender as explanatory variables. Reporting center was a categorical variable with nine levels and we set the public hospital in Karachi as the reference level. Due to its more central urban location we hypothesized that the victims presenting to this center would have the greatest health access and shortest travel times. In addition, it reported the largest number of cases therefore was a suitable center to select as a reference category for fitting the model. Pearson's chi-squared was used to test for goodness of fit. The test was appropriate as the model consisted mostly of non-unique covariate patterns [11]. Multiple regression analyses were performed with travel time to ER as the response variable, in order to assess differences in health access of patients following dog-bites with reporting center, age (centered at the mean) and gender as explanatory variables. During model validation, time data (recorded in minutes) were log-transformed for the residuals to approximate a normal distribution. Robust regression using bootstrapping for 95% confidence intervals was utilized in order to take into account the effect of outliers, but these were not excluded from the analysis as they may represent cases that were travelling to centers from distant locations and provide valuable information with regards to medical access.

### Spatial Analysis

Spatial analyses were limited to the city of Karachi as it had the highest number of cases and administrative boundaries for the city (as distinct town units) were easily available to permit geocoding of addresses. In addition, an insufficient number of addresses in other cities were deemed appropriately detailed for inclusion in spatial analysis, or were outside city limits. Due to the presence of a large stray dog population and wide variations in social determinants of health such as poverty and homelessness we hypothesized a non-uniform distribution of dog-bite incidence in the city [12]. As a predominantly Muslim country, dog-ownership is quite low in Pakistan. Stray-dogs are frequently encountered in Karachi, particularly in low-income areas and are therefore less likely to be vaccinated [12]. Each patient's address was manually geocoded to an administrative town in Karachi as most respondents did not know the zip (postal) codes for their house address for automated matching to administrative towns. Counts were aggregated up to area-levels for each of the administrative towns and were utilized to generate choropleth maps using ArcGIS (ESRI 2011. ArcGIS Desktop: Release 10. Redlands, CA: Environmental Systems Research Institute). Population estimates were obtained from the last available census carried out in the city in 1998 using a uniform annual population growth rate of 3.5% for each town up to the year of the study [13]. This growth rate accounts for both natural growth of population as well as increases due to migration into the city. Distribution of dog-bites was examined for spatial dependence and clustering of cases using the Moran's I test statistic, a global index for spatial autocorrelation that compares differences in counts amongst contiguous area-units after adjusting for population in the overall dataset. We used a Poisson regression, with counts of bites as the response variable and town as the explanatory variable, to indicate whether there was spatial dependence between towns. Population estimates were log transformed and used as an offset for the two years to assess differences in incidence rate ratios between individual towns. Risk estimates of dog-bites for each of Karachi's 18 towns were calculated using the ratio of observed cases to the expected (based on total population) to identify towns with increased risk of exposure. A spatial cluster detection analysis for increased rates was subsequently carried out using a discrete Poisson model, with the maximum spatial cluster size as <50% of the population (on SaTScan version 8.0) to identify primary and secondary clusters with increased rates [14], [15].

The costs of the mHealth surveillance system have been described in US Dollars and International US Dollars (I$), a conceptual currency which adjusts costs for purchasing power parities between countries. The costs have been calculated from a health services perspective and do not include patient related variables such as lost productivity.

## Results

### Patient Characteristics

A total of 6,470 cases of animal-bites were reported during the 24 months of surveillance, of which 96% (6,212) were due to dog-bites. The largest number of cases was reported at the public tertiary care hospital in Karachi (48.26%), as shown in Table 1. Females accounted for only 20.6% of the total and their proportions remained low after adjusting for centers. The median age for cases was 20 years (inter-quartile range, 10–35) and 38.9% of cases were under 15 years of age. Using the WHO classification system for severity of dog bites with Category III being the most severe, 40% of patients had Category I (least severe) bites, 28.1% had Category II bites and 31.9% had Category III bites. Following adjustment for center, two public hospitals in Bahawalpur and Karachi reported a greater proportion of Category I bites, where as the other centers reported greater numbers of Category III bites. Less than 1% of cases reported receiving pre-exposure vaccination across all study sites.

**Table 1:** Characteristics of dog-bite victims identified through screening at nine emergency rooms in Pakistan (Feb 2009–Feb 2011).

	Karachi Public Hospital	Karachi Private Hospital	Hyderabad	Thatta	Abottabad	Manshera	Peshawar	Quetta	Bahawalpur	Total
**City Population**		17,205,330	2,078,367	44,409	1,430,238	70 293	1,439,205	896,090	543,929	23,593,159
**Population Type**	Urban	Urban	Mixed	Rural	Rural	Rural	Mixed	Mixed	Mixed	
**Total Cases**	2,998	711	708	58	133	231	817	115	220	6,212
**(%)**	(48.26)	(11.45)	(11.40)	(0.93)	(2.14)	(3.72)	(13.15)	(1.85)	(7.10)	
Male	2,479	578	575	56	123	150	650	103	220	4934
(%)	(82.69)	(81.29)	(81.21)	(96.55)	(92.48)	(64.94)	(79.56)	(89.57)	(49.89)	(79.43)
Female	519	133	133	2	10	81	167	12	221	1,278
(%)	(17.31)	(18.71)	(18.79)	(3.45)	(7.52)	(35.06)	(20.44)	(10.43)	(50.11)	(20.57)
Median age	22	20	18	17	20	20	13	20	20	20
(IQR)	(11–35)	(9–36)	(10–35)	(15–20)	(12–50)	(11–35)	(8–27)	(10–29)	(10–40)	(10–35)
Children <15	1,011	283	302	10	43	96	447	48	120	2,360
(%)	(34.54)	(40.60)	(43.90)	(19.61)	(34.13)	(42.29)	(55.81)	(42.11)	(27.91)	(38.94)
**WHO Bite Category**										
I	1,810	16	35	2	0	1	13	5	246	2128
(%)	(71.09)	(2.38)	(4.94)	(3.45)	(0.00)	(0.48)	(2.38)	(4.35)	(71.72)	(40.02)
II	616	199	301	3	31	38	217	15	74	1494
(%)	(24.19)	(29.66)	(42.51)	(5.17)	(26.05)	(18.10)	(39.67)	(13.04)	(21.57)	(28.10)
III	120	456	372	53	88	171	317	95	23	1695
(%)	(4.71)	(67.96)	(52.54)	(91.38)	(73.95)	(81.43)	(57.95)	(82.61)	(6.71)	(31.88)

*Abbreviations: IQR, Inter-Quartile Range; WHO World Health Organization.*

*Source of city populations:* http://world-gazetteer.com/wg.php?x=&men=gcis&lng=en&des=wg&geo=-172&srt=pnan&col=abcdefghinoq&msz=1500&va=&pt=a.

### Estimation of Dog-Bites in Pakistan

A previous study from a major public-sector tertiary care hospital in Karachi estimated that 25–35% of cases in the city visited its facility for management of dog-bites, [7]. The estimation was based on interviews with hospital staff and the hospital's proportion of total vaccines consumed in the city. The ERs included in this study were tertiary care referral centers of similar size therefore we used the total number of cases notified and the same estimated range of the proportion of dog-bites reported to extrapolate the incidence of dog-bites in the catchment areas of these centers. For the two centers in Karachi this equates to 8,565 to 11,992 (2,998/0.25 to 2,998/0.35) dog-bite cases for the public hospital and 2,031 to 2,844 (711/0.25 to 711/0.35) for the private hospital. For the centers outside of Karachi this equates to a total of 7,151 to 10,012 (2,503/0.25 to 2,503/0.35) cases of dog-bites. The estimated range of dog-bites over the two-year period in the catchment area of these centers is therefore, 17,774 to 24,848. Based on the total populations where these centers are located the estimated incidence of dog-bites is 38 to 53 per 100,000 per year.

### Health-Seeking Behavior and Hospital Access Outcomes

Overall, a greater proportion of cases sought immediate healthcare at a non-medical facility or administered self-treatment (52.29%) (Table 2). The median time to ER was 30 minutes (IQR 5–1,800 minutes) and ranged from 5 to 7,800 minutes. A proportionately higher number of cases from the Karachi public hospital sought immediate care at medical facility (80.92%) whereas non-medical or self-treatment was more commonly reported for the remaining centers. Cases reporting from Hyderabad had the shortest median travel time to ER (20 minutes) where as those reporting from Mansehra had the highest (120 minutes).

**Table 2:** Patient health-seeking behavior following dog-bites and travel time to emergency rooms in Pakistan (Feb 2009–Feb 2011).

Reporting Center	Patient Health-Seeking Behavior		Patient Time to Emergency Room		
	Non-Medical or Self-Treatment	Medical	Median Time (minutes)	IQR (minutes)	Range (minutes)
Karachi Public Hospital	572	2,426	25	20–45	5–7,800
(%)	(19.08)	(80.92)			
Abottabad	73	60	100	60–120	5–1,145
(%)	(54.89)	(45.11)			
Hyderabad	436	272	20	15–30	7–1,020
(%)	(61.58)	(38.42)			
Quetta	85	30	60	20–180	10–1,680
(%)	(73.91)	(26.09)			
Karachi Private Hospital	598	113	30	20–60	4–1,800
(%)	(84.11)	(15.89)			
Thatta	52	6	60	30–60	10–300
(%)	(89.66)	(10.34)			
Mansehra	211	20	120	60–180	5–480
(%)	(91.34)	(8.66)			
Bahwalpur	428	13	30	10–60	5–360
(%)	(97.05)	(2.95)			
Peshawar	793	24	60	60–120	10–1800
(%)	(97.06)	(2.94)			
**Total**	**3,248**	**2,964**	**30**	**5–1,800**	**5–7,800**
**(%)**	**(52.29)**	**(47.71)**			

Table 3 describes results of the regression analyses of patient health-seeking behavior and travel time to ER. Patients from all other centers, relative to those visiting the public hospital in Karachi (reference level) were more likely to seek immediate healthcare at a non-medical facility, adjusted for age and gender (p<0.01). Peshawar and Bahawalpur were most likely to seek immediate health care at a non-medical facility or administer self-treatment compared to visiting a medical facility (adjusted odds ratio 144.45 and 131.36 respectively). These odds ratios imply that cases from Peshawar and Bahawalpur were over a hundred times more likely to seek immediate care at a non-medical facility as compared to the public hospital in Karachi. Excluding Karachi, cases from Abbottabad (adjusted odds ratio 5.12) and Hyderabad (adjusted odds ratio 6.87) were relatively less likely to seek immediate care at a non-medical facility or administer self-treatment as compared to the remaining centers. Cases reporting to the Hyderabad center had the shortest travel time to the ER compared to the rest of the centers, while those at Mansehra and Abbottabad had the longest travel times. Age and gender were not significantly associated with health-seeking behaviors or travel times to ER.

**Table 3:** Results of multiple logistic regression analysis of dog-bite patient health-seeking behavior and multiple linear regression analysis of travel times to emergency rooms in Pakistan (Feb 2009–Feb 2011).

	Patient Health-Seeking Behavior			Patient Time to Emergency Room		
Explanatory Variable	OR*	95% CI†	p-value	β†	95% CI	p-value
Center						
Karachi Public Hospital (reference)	-			-		
Abbottabad	5.12	3.59–7.30	<0.01	1.14	1.03–1.27	<0.01
Hyderabad	6.88	5.76–8.22	<0.01	−0.39	−0.44–0.39	<0.01
Quetta	12.32	8.03–18.90	<0.01	0.48	0.10–0.85	<0.01
Karachi Private Hospital	22.57	18.09–28.15	<0.01	0.04	−0.04–0.11	0.24
Thatta	38.79	16.56–90.83	<0.01	0.61	0.39–0.84	<0.01
Mansehra	44.29	27.72–70.77	<0.01	1.32	1.26–1.41	<0.01
Bahawalpur	131.36	74.91–230.35	<0.01	0.30	0.18–0.43	<0.01
Peshawar	144.45	95.16–219.26	<0.01	1.06	1.01–1.14	<0.01
Age	1.00	1.00–1.01	0.01	0.0002	−0.001–0.001	0.71
Gender‡	1.15	0.97–1.38	0.11	0.04	−0.05–0.05	0.86
Intercept	0.23	0.20–0.25		3.49	3.46–3.52	<0.01
Pearson's χ^2^	6.30		0.62			

* An Odds Ratio of greater than 1 indicates individuals were more likely to seek immediate care at a non-medical facility or self-treatment compared to visiting a medical facility.

† Represent coefficients from multiple linear regression model.

‡ Male reference level.

Overall 13 cases died due to rabies at an Emergency Room, 8 of which took place in Mansehra. Only one case was confirmed using the Fluorescent Antibody Test (FAT) on post-mortem examination, with the remaining diagnoses based on clinical symptoms.

### Spatial Analysis of Dog-Bites in Karachi

**Figure 2:**
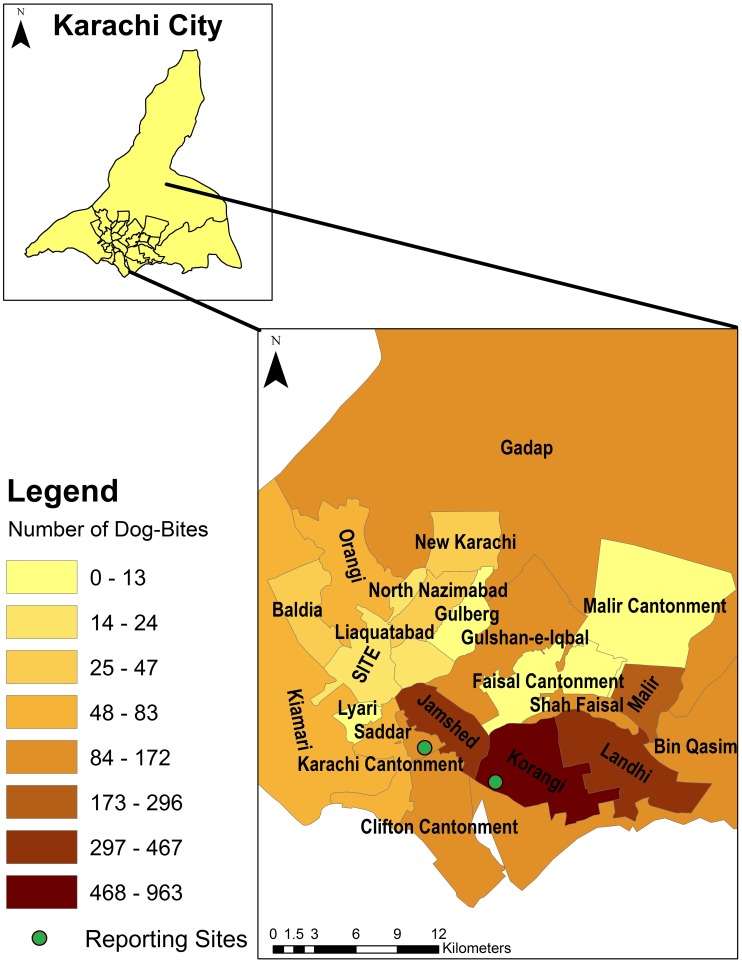
Spatial mapping of dog-bites cases by administrative towns in Karachi, Pakistan between Feb 2009–Feb 2011.

**Figure 3:**
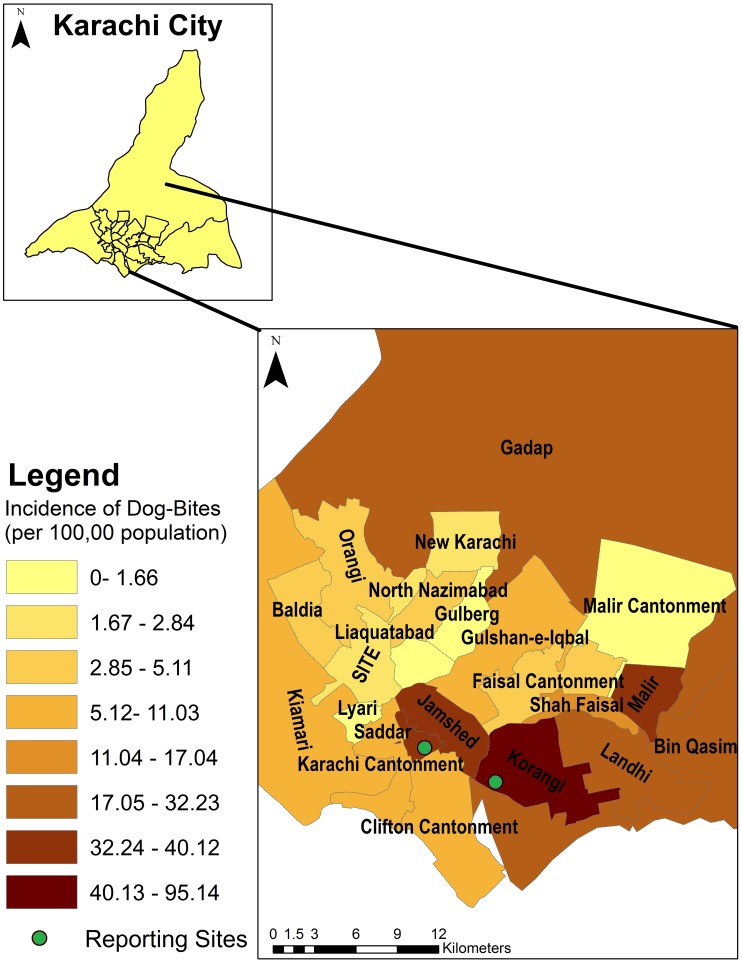
Incidence of dog-bite cases per 100,000 by administrative towns in Karachi, Pakistan between Feb 2009–Feb 2011.

**Figure 4:**
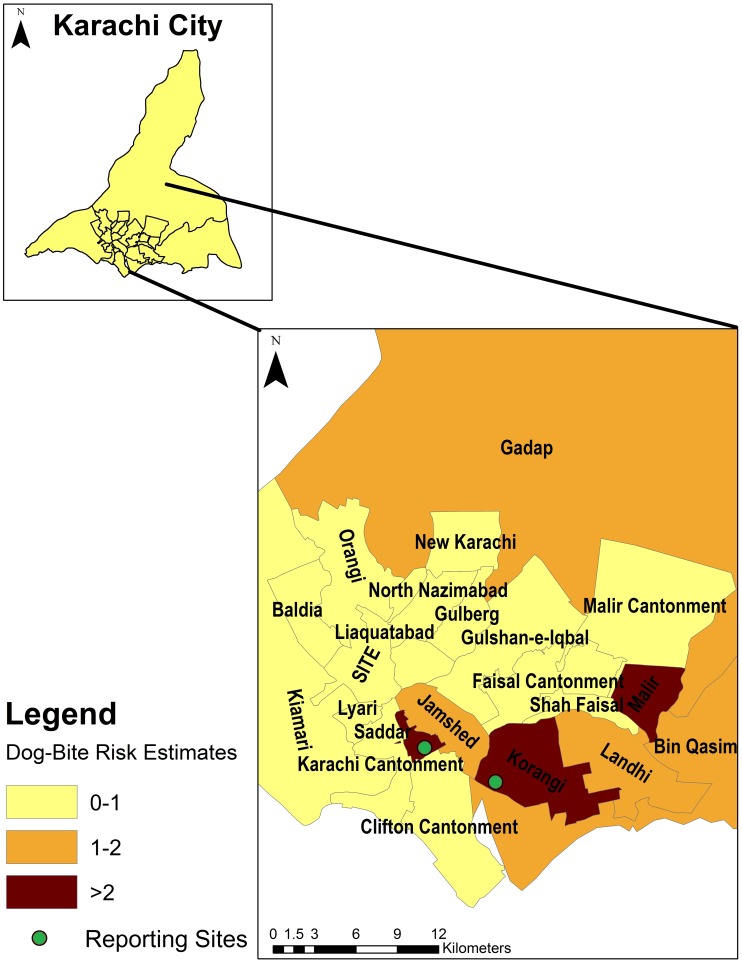
Risk estimates of dog-bites by administrative towns in Karachi, Pakistan between Feb 2009–Feb 2011.

**Figure 5:**
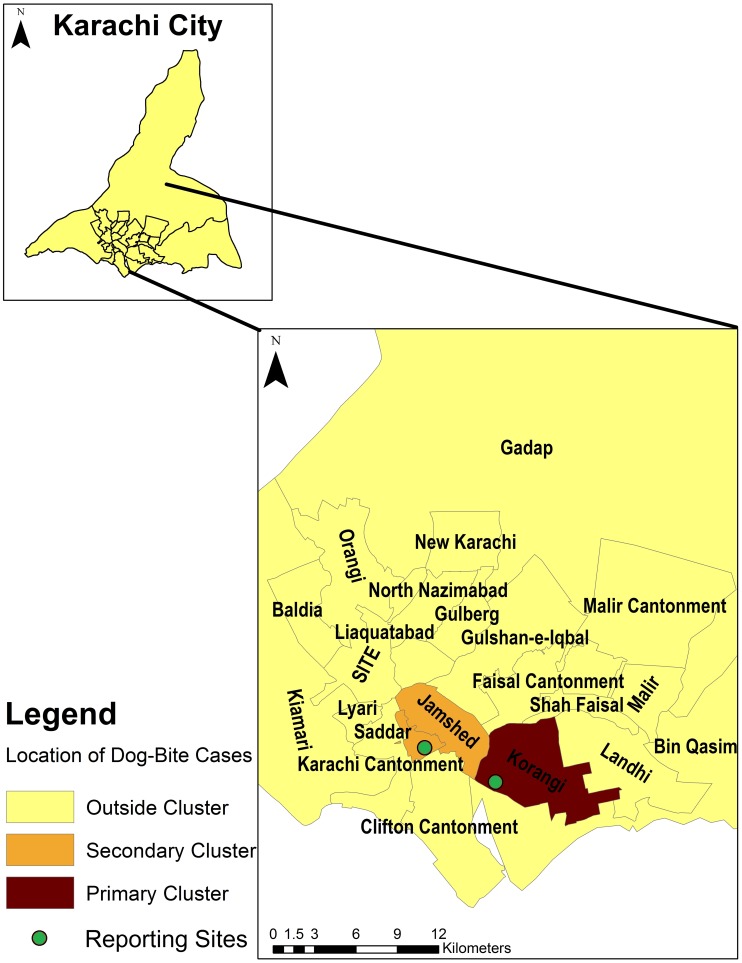
Cluster analysis of dog-bites cases by administrative towns in Karachi, Pakistan.

Figure 2 displays aggregated counts of dog-bite cases for the city of Karachi. Korangi, Jamshed, Landhi and Malir had the highest number of cases of dog-bites. Figure 3 displays incidence rates of dog-bites in the city. The incidence rates are higher in the central towns of Korangi, Malir and Jamshed, although high rates were also observed in the more peripheral towns (Bin Qasim and Gadap). The Moran's I index for spatial autocorrelation was 0.20, statistically significant for spatial clustering of cases (p-value<0.01). There were significant differences in incidence rates between administrative units (towns) based upon Poisson regression analyses, further suggestive of spatial dependence. Figure 4 shows the highest risk estimates for dog-bites in Korangi, Malir and Karachi Cantonment, although Landhi, Jamshed, Gadap and Bin Qasim also had risk estimates of greater than 1. Cluster analysis (Figure 5) identified Korangi as the primary cluster with a relative risk of 7.14 (log likelihood ratio 942.16, p-value<0.01). Karachi Cantonment (log likelihood ratio 91.95, p-value<0.01) and Jamshed (log likelihood ratio 35.08, p-value<0.01) were identified as secondary clusters, each with a relative risk of 2.10. These three towns of Karachi represent contiguous area-units where a clustering of high dog-bite rates was observed.

### Cost of Mobile-Phone Based Surveillance System

As described in Table 4, our experience highlights low deployment and operations costs for our mHealth system across a wide geographic region. The total direct costs of equipment and staffing for two years of surveillance at 8 sites was USD 44,408 with approximately USD 19,385 for capital costs and USD 25,023 for salary support and other operational costs. We estimated the cost per case detected was USD 7.15 (I$ 17.26). The average cost per center was USD 4,934 per center enrolled. Costs did not vary significantly between centers and differences were largely attributable to travel expenses for management and program staff for on-site trainings.

**Table 4:** Costs of mobile phone based dog-bite and rabies surveillance system implemented at nine emergency rooms in Pakistan, Feb 2009–Feb 2011.

	Costs (USD)*	Number	Costs (International USD)†
**Capital Costs**			
Equipment (GSM Cellphones)	3,500	14	3,500
Equipment (Server)	3,000	01	3,000
**Implementation Costs**			
Staff Training	6,100	13	16,203
Transportation (Air)	4600	-	12,218
Transportation (Ground)	2185		5,803
**Running Costs**			
Management Salaries	11,855	02	31,489
Field Staff Salaries	10,253	11	27,234
Accounts	615	01	1,633
Airtime charges	600	-	1,593
Others Operational Costs	1,700	-	4,515
**Total**	44,408		107,188

* Costs described in US Dollars based on 2009 exchange rates (1 USD = 85.19 Pakistani Rupees).

† Costs described in 2009 purchasing power parity exchange rates (1 International USD = 32.30 Pakistani rupees). The conversion rates use price levels in the United States as reference, which means one International Dollar has the same purchasing power as one US Dollar in the United States. Equipment costs have been assumed to be uniform internationally.

## Discussion

The high costs associated with establishing and maintaining infectious disease surveillance systems makes poor countries such as Pakistan vulnerable to outbreaks and hinders control of endemic diseases. In the case of rabies, whose transmission is primarily mediated through a canine reservoir, understanding and mitigating the risk of exposure to dog-bites provides an opportunity to control a universally fatal disease [10].

No prior studies from Pakistan have attempted to quantify the burden of dog-bites and rabies in the country as multi-center surveillance was absent in the country. Published studies from the region have focused largely on clinical evaluations of dog-bite cases and wound-management practices. In addition, such studies have relied upon retrospective data or historical recall to ascertain bite-exposure with inherent limitations of such approaches, in particular with missing patient data for risk-factors and outcomes. Our mHealth system permitted electronic data capture at large public and private facilities, and allowed for the prospective assessment for a range of risk factors related to dog-bites and documentation of patient mortality. Through this study we highlight a large, previously undocumented burden of dog-bites in Pakistan. Given the challenges in setting up such surveillance mechanisms, the absence of well-documented evidence of this disease burden has prevented adequate control mechanisms to take root in the country, even though such initiatives began several decades earlier [16]. Secondly, we demonstrated the application of mHealth technologies as a novel tool to overcome challenges in setting up effective surveillance systems in developing countries through a large-scale, nationwide implementation. Public officials are often hesitant to introduce innovation within programs and the absence of studies from Pakistan describing the utilization of such technologies has limited their wider adoption. By describing a range of analyses possible through prospective electronic data capture and its cost-effectiveness, this study may help advocate the use of mHealth & eHealth in disease surveillance amongst policy-makers and public officials over conventional paper-based methods for infectious disease surveillance.

This study identified large variations in patient access to care and health-seeking behaviors across different reporting sites that may serve as indicators of risk for developing rabies. Cases presenting to urban centers such as Karachi and Hyderabad were least likely to visit non-medical facilities first or administer self-treatment and unsurprisingly had shorter travel times compared to centers serving more rural populations. An exception to this was Abbottabad where cases were less likely to seek care at a non-medical facility compared to other centers, although the median travel time to the ER in Abbottabad was significantly longer than in Karachi and Hyderabad. This likely reflects a more educated and affluent peri-urban and rural population accessing the ER at Abbottabad than at some of the other reporting sites. Similarly, the large catchment population served by the private hospital in Karachi that includes many rural fishering communities could have accounted for the differences in health-seeking behavior seen between the two reporting ERs in the city. A disproportionately high number of deaths were reported at the ER in Mansehra, which serves as a referral center for remote, rural mountainous communities. This center reported the longest adjusted travel times and it is likely that these cases presented when symptoms had already developed. In addition, the absence of cell-culture vaccines at this center may have also contributed to the higher mortality.

A far greater proportion of males presented to ERs as compared to females, likely a reflection of total time spent outdoors, and possibly associated reduced risk of exposure, but this could also reflect limited access to health facilities for females. Women in Pakistan are less frequently outdoors, particularly in urban areas and non-agricultural rural communities and are also likely to have less access to healthcare compared to males. Although children were frequently victims of dog-bites, the age distribution of cases was not necessarily suggestive of increased risk given the young age of the Pakistani population. We found that at most centers, there were substantially more patients with severe dog-bite wounds (Category III). Possibly, less severe bites were being treated at lower levels of care, including using non-allopathic providers or self-care.

Several public health initiatives will be required through evidence generated from this study. Foremost, advocacy efforts for rabies control need to be greatly strengthened and supported. It is important to emphasize that appropriate management of dog-bites and control of rabies is not neglected by policy-makers, public officials and hospitals given the focus on other key infectious diseases programs such as polio eradication, measles elimination and tuberculosis control. Significant investments will need to be made to improve public awareness about appropriate care following dog-bite exposure particularly in rural areas. Services for post-exposure prophylaxis need to be greatly expanded beyond urban centers. This may involve setting-up dog-bite care clinics within the publicly administered Basic Healthcare Units system or increasing the availability of reduced costs rabies vaccines in the private sector to promote its uptake in less developed regions of the country. Pre-exposure vaccination for low-income workers outside of more formalized animal farming settings such as abattoirs also needs to be considered by the government. Larger public facilities, especially those in more remote areas could benefit from intra-dermal injections of cell-cultured vaccines that are less costly and requires less vaccine therefore reducing the possibilities for potential stock-outs.

Limited efforts under the banner of rabies control currently exist in most cities of Pakistan. In Karachi, measures largely include shooting of stray-dogs and depositing large numbers on an uninhabited island in order to help control their population. Spatial analyses can help direct more effective rabies control initiatives such as canine vaccination in low-resource settings by identifying high-risk populations. The presence of a large abattoir on the eastern border of Landhi, may have attracted large stray dog-populations in the area and contributed to the high incidence of reported cases from the immediately adjacent and more densely populated, low-income towns of Korangi and Malir. Our experience of establishing public health services and community cohorts in these towns spanning the past several years supports the premise of a large stray dog population concentrated in the area though their numbers are as yet not quantified. Based on the findings from the spatial analysis, we have approached veterinary-physicians in the military (which administers several areas of the city) as well as officials in the city municipal offices to establish canine vaccination efforts focused around the eastern parts of the city highlighted within the maps as well as in Gadap Town, a large urban sprawl towards the north-east. Such programs would need to involve close coordination between administrative authorities of each town and can be supported in data monitoring and evaluation by academic organizations. Investigation of urban canine habitat and migration patterns will be required that can further help narrow areas with high risk of exposure and these activities can also be supported through the use of mobile-phone technologies. In addition, a more detailed understanding of dog-ownership and proportion of stray-dogs in high risk areas will be required. Findings from these investigations can help determine resource requirements and allow for targeted immunization campaigns.

A major limitation of the system was the small number of centers that were enrolled in the study relative to all facilities where dog-bite cases might access care. This may lead to selection bias while making inferences about population characteristics. It is difficult to quantify the extent of missing data since dog-bite victims could visit any one of a number of public or private healthcare facilities, including non-allopathic providers across the country or may not seek any treatment at all. Resource constraints required selection of a limited number of high-volume tertiary care facilities that were also regional referral centers for dog-bites. Our estimates of the total number of dog-bites cases in the country are therefore likely to be conservative since fewer number of notifications may have taken place from rural centers with wider catchment areas as well as notifications from outside our reporting sites. For patient health access and health-seeking behaviors including centers within the regression models may have helped account for area-level indicators such as land use and transportation. However, socio-economic indicators from patients were not collected as part of the questionnaire and could therefore not be included. Patient travel time to ER was therefore used to approximate access to care.

The spatial analysis included population estimates that were extrapolated from census data that were more than a decade old. A uniform population growth rate for this entire period and across all of Karachi's administrative units may not accurately reflect higher population growth in recent years and increases due to migration which may be more concentrated in certain areas. The accuracy of incidence rates for dog-bites is likely to be most sensitive to this variation in population; however, identification of clustering may be less affected since it is more dependent on the distribution of cases. Other well-known risk factors for dog-bites such as poverty, homelessness and dog population densities were unavailable or not generalizable, and prevented a more robust analysis. Investigating covariates that might account for some of the observed spatial dependence could identify potential risk factors for dog-bites and can further aid in the decision-making process. Since only two centers were enrolled in Karachi reporting bias may have contributed towards the observed clustering of cases around Korangi, a major limitation of the spatial analysis. Increasing the number of surveillance sites would have accounted for this. Employing case-control methodologies with mapping of point-pattern data in order to more appropriately quantify differences in risk could help enhance inferences regarding the spatial distribution of dog-bites.

Scale-up and sustainability are major limitations that prevent this system from being more effective. Whilst short, on-site trainings provided sufficient familiarity for screeners to utilize the software for reporting, the data-collection process itself was occasionally perceived as disruptive to patient-care. This suggests that any integration of innovative technologies within a health system needs to take into account the broader environment in which it will operate. Health administration in South-Asia, particularly in the public sector is frequently hierarchical and scaling-up such initiatives is challenging without support from senior officials, as was the case at two sites we had approached. Data collected via the system may be integrated within wider reporting systems. OXD stores data as a relational database that allows data export in CSV formats for data integration provided other systems support such formats. For non-supportive systems programs would be required to appropriately transform the data. Technological challenges in scale-up of the system infrastructure were largely related to data-storage capacity of servers. Mobile internet connectivity was not an issue at our reporting sites largely due to rapid advances in telecommunications coverage over the past decade. However, it may be a challenge to extend this system to more remote settings with poor connectivity. Newer technologies such as 3G and O3B (Other 3 Billion) that are under development in the country may help improve connectivity and speed of data-transfer. Limitations in the software mobile-application utilized for the surveillance system included the absence of SMS reminders for vaccinations. Phone calls attempted by screeners to follow-up were not logged in the database, and hence did not generate automatic alerts for patients potentially defaulting their vaccine schedule.

Assessing the cost-effectiveness of this system is challenging in the absence of any mechanisms for surveillance of dog-bites and rabies in Pakistan. Our cost estimate of USD 7.15 per dog-bite case identified is higher compared to other mHealth surveillance evaluations in resource-limited settings [17]–[19]. Such studies are also not directly comparable, given their focus on mass-screening and the relative infrequent occurrence of dog-bites compared to epidemic-prone infectious diseases that are usually monitored by such surveillance systems. Capital and operating costs to implement and sustain surveillance systems for neglected diseases often prove prohibitive for government agencies. Our experience suggests that contrary to general perceptions, they do not require large financial investments and costs per case identified are likely to decline over time as surveillance is extended due to returns on fixed costs with more cases detected and if more centers are added to the system. In smaller facilities such as private family physician clinics where the number of cases presenting with dog-bite will be low, it may be more cost-effective to develop SMS based reporting systems where providers are incentivized to report cases and the submitted information is integrated with the main database. In addition, growth of the Smartphone industry in recent years has greatly reduced prices and mobile-phones with similar functionality can now be purchased for approximately USD 100, a process that can be facilitated through bulk-purchase from vendors and reduced data-transfer rates by telecom operators.

Sustainability of such surveillance systems are generally linked to funding commitments from governments or donor agencies. Through this study we attempted to first highlight the need for dog-bites and rabies surveillance and secondly demonstrate the ability of mHealth technologies to deliver such a system. However, the type of analyses carried out in this study may well be beyond the scope of screeners or even public health administrators to carry-out on a routine basis, thus limiting its appeal. Enhancements to the software therefore need to include elements of product design utilized by commercial technology companies that involve sophisticated yet user-friendly server front-end applications for easier data-visualization (e.g. GIS) and presentation of complicated data analyses (e.g. spatial modeling). Costs associated with such software development was not feasible within the project funding. An alternative sustainability strategy may therefore be to seek investor funding that helps further develop the software with a commercial intent and for it to be delivered as a package to provincial or national governments. The business model could involve recovering costs by charging for each case registered and tracked via the system, rather than seeking upfront costs for project implementation which may be met with greater resistance.

### Conclusion

We identified a high burden of severe (World Health Organization category III) dog-bites and substantial variation in patient health-seeking behavior and access to care across different regions of Pakistan. Spatial analyses were suggestive of clustering of dog-bite cases in Karachi and can facilitate preventive efforts. Low-cost mobile phone technologies have the potential to address gaps in surveillance systems in low-resource settings. Further cost-effectiveness studies are required of large-scale implementations of mHealth based infectious diseases surveillance systems in developing countries to generate evidence for necessary resources for scale-up and sustainability.
